# Editorial: Psychoneuroendocrinology of psychosis disorders, volume II

**DOI:** 10.3389/fpsyt.2024.1398841

**Published:** 2024-04-03

**Authors:** Alexandre González-Rodríguez, Alexis E. Cullen, Mary V. Seeman

**Affiliations:** ^1^ Department of Mental Health, Mutua Terrassa University Hospital, Centro de Investigación Biomédica en Red de Salud Mental (CIBERSAM), University of Barcelona, Terrassa, Spain; ^2^ Division of Insurance Medicine, Department of Clinical Neuroscience, Karolinska Institutet, Solna, Sweden; ^3^ Department of Psychosis Studies, Institute of Psychiatry, Psychology & Neuroscience, King’s College London, London, United Kingdom; ^4^ Department of Psychiatry, University of Toronto, Toronto, ON, Canada

**Keywords:** psychoneuroendocrinology, psychosis, psychosocial stress, thyroid, perinatal

Recent research on psychopathological pathways has uncovered the important role of hormones. Epidemiological evidence suggests that endocrine disorders (e.g. hypo- and hyperthyroidism, Cushing’s syndrome, gestational diabetes, polycystic ovaries, Turner’s disease) all increase the risk of psychosis and that altered hormone levels (e.g. sex hormones, glucocorticoids, thyroid hormones) influence the clinical course and prognosis of psychotic illness (1–3). While it is well known that the treatment of psychotic disorders consists mainly of anti-dopaminergic drugs and that changes in neurotransmitters must, therefore, be involved in disease etiology, it is less commonly acknowledged that these neurotransmitter systems are influenced by upstream endocrinological circuits (4).

The aim of this Research Topic was to present the most recent and relevant advances in our understanding of the role of hormonal factors implicated in the etiopathology of psychotic disorders. Five studies are included in this Research Topic. Two explored the relationships between thyroid function, structural and functional biological mechanisms, and clinical symptoms of psychosis and schizophrenia (Sumi et al.; Toll et al.). One study investigated the impact of circulating neuropeptides on cognitive function and symptoms in schizophrenia and related disorders (Yu et al.). The concept of stress is closely related to endocrine disruption. One study investigated how psychosocial stress in particular influences the levels of cerebrospinal fluid lactate dehydrogenase in psychosis (Giné-Servén et al.). Postpartum psychosis has always been suspected to result from the dramatic changes in maternal estrogen and prolactin levels after delivery. This topic was investigated by (Palacios-Hernández et al.).

Sumi et al. provided a case report of a 32-year old woman with schizophrenia who developed Graves’ disease. She presented with symptoms of agitation, speech disorder, and auditory and visual hallucinations that only appeared in the context of a thyroid storm. Thyroxine levels were positively associated with the severity of psychotic symptoms. She was treated with methimazole and dexamethasone and paliperidone (oral and depot) were initiated to reduce psychotic symptoms. This case report emphasizes the importance of monitoring thyroid function in patients suffering from schizophrenia, perhaps especially in those who present with visual hallucinations, which are relatively rare in the majority of forms of schizophrenia.

Toll et al. also examined thyroid hormone levels, in addition to peripheral brain-derived neurotrophic factor (BDNF), and hippocampal volume, this time in 50 antipsychotic-naïve first-episode psychosis (FEP) patients. In total, thirty-six percent of the sample had altered thyroid-stimulating hormone (TSH) levels, part of which (32%) were elevated. The results showed that TSH levels were negatively correlated with both peripheral BDNF and hippocampal volume. The authors concluded that thyroid hormone may play a neuroprotective role with respect to the hippocampus in FEP by increasing BDNF concentrations. These hormonal markers have been poorly investigated in this population.

The association of plasma neuropeptides with clinical symptoms and cognition was investigated in 54 individuals experiencing their first episode of schizophrenia, 52 bipolar disorder patients, 35 patients with major depressive disorder and 54 non-psychiatric controls (Yu et al.). The results show that α-MSH, neurotensin, orexin-A, oxytocin, and substance P were decreased in all three patient groups compared to the control group. Lower oxytocin levels and higher levels of substance P in this study were associated with greater severity of psychotic symptoms in both schizophrenia and bipolar disorder. β-endorphin was found to be correlated with early morning wakening in all patient groups. Plasma neuropeptides were consequently identified as potentially important transdiagnostic biomarkers of severe mental illness.

While there are many forms of stress, psychosocial stress is often not investigated in biological studies that focus on cerebrospinal fluid (CSF). Following up on the gaps in this field, Giné-Servén et al. investigated whether psychosocial events mediate/moderate the association between duration of untreated psychosis (DUP), lactate dehydrogenase levels in CSF and other CSF biomarkers in FEP patients. Among 95 individuals with FEP, 56.8% reported a stressful life event in the previous 6 months. Among these, a shorter DUP was correlated with LDH concentrations after adjustment for potential confounding factors. It is suggested that psychosocial stress modulates the association between CSF biomarkers and the onset of psychosis.

Palacios-Hernández et al. provided a review of hormonal changes influencing the onset of perinatal psychotic disorders and cognitive dysfunction in perinatal women. In total, fourteen studies were included in the review, which mainly tested the following five hypotheses: 1) change in dopamine receptors, 2) increase in cortisol levels, 3) change in estrogen levels, 4) determination of thyroid dysfunction and 5) decrease in oxytocin levels. Only one report studied the relationship between hormones and cognition. Evidence for three of the examined hypotheses in the review was found: 1) increased dopaminergic response was related to high sensitivity of hypothalamic receptors, 2) elevated cortisol levels were observed, in line with the neural diathesis-stress model of psychosis, and 3) a relationship was found between thyroid dysfunction and psychotic episodes. There was no relationship between hormones and cognitive function.

To summarize what was learned in this special Frontiers Research Topic investigating endocrinological effects on psychosis in 2024, we first emphasized the emerging role of the thyroid gland on brain structure and function in patients with early schizophrenia (5). We also learned that neuropeptides, although not identical to substances generally considered hormones, are important inter-cellular signaling molecules whose levels differ significantly between patients with severe mental illness and controls as a result of psychosocial stress. Finally, with regard to perinatal psychotic disorder in which a relationship between hormones and psychotic symptoms has previously been shown, we learned that the question of hormonal influence on cognition in this disorder remains understudied and uncertain.

## Author contributions

AG-R: Conceptualization, Writing – original draft, Writing – review & editing. AC: Writing – review & editing. MS: Supervision, Validation, Writing – review & editing.

## References

[1] De Leo S Lee SY Braverman LE. Hyperthyroidism. Lancet. (2016) 388:906–18. doi: 10.1016/S0140-6736(16)00278-6 PMC5014602 27038492

[2] Abramova O Zorkina Y Ushakova V Zubkov E Morozova A Chekhonin V. The role of oxytocin and vasopressin dysfunction in cognitive impairment and mental disorder. Neuropeptides. (2020) 83:102079. doi: 10.1016/j.npep.2020.102079 32839007

[3] Rutigliano G Chaumette B Seeman MV. Editorial: Psychoneuroendocrinology of psychosis disorders. Front Psychiatry. (2020) 11:607590. doi: 10.3389/fpsyt.2020.607590 33240137 PMC7680761

[4] Sonnenschein SF Gomes FV Grace AA. Dysregulation of midbrain dopamine system and the pathophysiology of schizophrenia. Front Psychiatry. (2020) 11:613. doi: 10.3389/fpsyt.2020.00613 32719622 PMC7350524

[5] Misiak B Stańczykiewicz B Wiśniewski M Bartoli F Carra G Cavaleri D. Thyroid hormones in persons with schizophrenia: a systematic review and meta-analysis. Prog Neuropsychopharmacol Biol Psychiatry. (2021) 111:110402. doi: 10.1016/j.pnpbp.2021.110402 34274416

